# Effects of Dietary Non-Fibrous Carbohydrate (NFC) to Neutral Detergent Fiber (NDF) Ratio Change on Rumen Bacteria in Sheep Based on Three Generations of Full-Length Amplifiers Sequencing

**DOI:** 10.3390/ani10020192

**Published:** 2020-01-22

**Authors:** Xuanxuan Pu, Xuefeng Guo, Khuram Shahzad, Mengzhi Wang, Chenyu Jiang, Junfeng Liu, Xiuping Zhang, Sujiang Zhang, Long Cheng

**Affiliations:** 1 College of Animal Science, Tarim University, Alar 843300, China; pxxlxl@126.com (X.P.); JCY2047@163.com (C.J.); ljfdky@126.com (J.L.); zxpdky@126.com (X.Z.); zsjdky@126.com (S.Z.); 2 Key Laboratory of Tarim Animal Husbandry Science and Technology of Xinjiang Production and Construction Group, Alar 843300, China; 3 Department of Biosciences, COMSATS University Islamabad, Park Road, Islamabad 45550, Pakistan; khuramsb@gmail.com; 4 College of Animal Science and Technology, Yangzhou University, Yangzhou 225009, China; 5 Faculty of Veterinary & Agricultural Sciences, Dookie Campus, The University of Melbourne, Melbourne, VIC 3647, Australia; long.cheng@unimelb.edu.au

**Keywords:** ruminal microbes, NFC/NDF ratio, amplifier sequencing

## Abstract

**Simple Summary:**

Rumen microbes play an important role in the health and production of ruminants, and they are influenced by dietary changes. In our study, we investigated the change of rumen bacteria under the four treatments of dietary non-fibrous carbohydrate (NFC) to neutral detergent fiber (NDF) ratios in sheep using three generations of full-length amplifiers sequencing. As rumen is a complex organ, and the effects of dietary NFC/NDF ratio change on ruminal bacteria might change over time, thus the study was conducted for four periods of 72 d in total. The results showed that the composition of rumen bacteria changed with different dietary NFC/NDF ratio during the experimental periods. Rumen bacterial diversity was decreased in dietary NFC/NDF ratio of 1.90 with the prolong of experimental periods. The main dominant phyla in Karakul sheep rumen didn’t change, while their relative abundance changed with dietary NFC/NDF ratio and experimental periods. The relative abundance of unidentified-*Lachnospiraceae* and main cellulose-degrading bacteria was higher in dietary NFC/NDF ratio of 1.37 than other groups (NFC/NDF ratio of 0.54, 0.96 and 1.90).

**Abstract:**

The study was conducted to investigate the effects of dietary NFC/NDF ratio change on rumen bacteria in sheep. Twelve Karakul sheep were assigned randomly into four groups fed with four dietary NFC/NDF ratios of 0.54, 0.96, 1.37, and 1.90 and they were assigned into groups 1, 2, 3, and 4, respectively. The experiment was divided into four periods: I (1–18 d), II (19–36 d), III (37–54 d), and IV (55–72 d). In each period, the first 15 d were used for adaption, and then rumen fluid was collected for 3 d from each sheep before morning feeding. The fluid was analyzed with three generations of full-length amplifiers sequencing. Results showed that the bacterial diversity of group 4 was decreased in period III and IV. At the phylum level, Bacteroidetes (37–60%) and Firmicutes (26–51%) were the most dominant bacteria over the four periods. The relative abundance of Bacteroidetes, Firmicutes, Tenericutes, and Spirochaete changed with dietary NFC/NDF ratio change over the four periods, but there was no difference among groups over the four periods (*p* > 0.05). At the genus level, unidentified-*Lachnospiraceae* was the dominant genus, and its relative abundance in group 3 was high during the period I and III (*p* < 0.05). The relative abundance of *Mycoplasma* in group 4 was high in the period I and II (*p* < 0.05). The relative abundance of *Succiniclasticum* was high in group 2 of period II (*p* < 0.05). At the species level, the relative abundance of *Butyrivibrio-fibrisolvens* was found to be high in group 3 during periods I and III (*p* < 0.05). The main semi-cellulose-degrading bacteria and starch-degrading bacteria were low, and there was no significant difference among groups over four periods (*p* > 0.05). Taken together, the dietary NFC/NDF ratio of 1.90 decreased the diversity of bacteria as a period changed from I to IV. While the main phylum bacteria didn’t change, their relative abundance changed with the dietary NFC/NDF ratio change over the four periods. The most prevalent genus was unidentified-*Lachnospiraceae,* and its relative abundance was higher in dietary NFC/NDF ratio of 1.37 than other groups. Similarly, the main cellulose-degrading species was higher in the treatment of dietary NFC/NDF ratio of 1.37 than other groups.

## 1. Introduction

Rumen fermentation plays an important role in the growth and production of ruminants. Yang et al. [1] demonstrated that up to 80% of starch, 50% of fiber, and 60% of organic matter in the diet can be fermented in the rumen, and energy is produced in the meantime, which can be utilized by ruminants. Rumen fermentation largely depends on microbiota (bacteria, protozoa, eukarya, and archaea [2,3,4]). Rumen microbes can utilize nutrition by breaking down feed into volatile fatty acids, bacterial protein, and energy, whereas the host animal can utilize the production [5]. After a long-term adaptation and evolutionary process, rumen microbiota and the host have developed a symbiotic relationship to maintain the host’s health and productivity [6]. There are many factors, such as species, age, diet, feeding, management, etc., influencing rumen environment [7,8,9,10]. The forage to concentrate ratio is a major factor, which affects rumen bacteria. The manipulation of forage to concentrate ratios can lead to major changes in dietary carbohydrate and fiber content and their ratio, which may help to optimize rumen fermentation and support higher ruminant production performance [11,12,13]. Previous work showed that by increasing the dietary non-fibrous carbohydrate to neutral detergent fiber (NFC/NDF) ratio, microbial diversity and population could be altered [14]. However, the work only lasted for 15 days. There is limited research investigated the change of rumen bacteria with different diets over a long term [15,16]. As rumen is quite complex, the effects of dietary NFC/NDF ratio on ruminal bacteria might change over time. Therefore, it is important to conduct a long term study to understand the microbial changes over a long period of feeding. In our study, we hypothesized that different dietary NFC/NDF ratio changes would affect rumen bacteria population and diversity, and these effects might change over time. With the development of technology, three generations of full-length amplifiers sequencing improves the resolution of species identification and the accuracy of microbial species composition identification in the samples [17,18]. Therefore, this study aimed to explore the effects of dietary NFC/NDF ratio change on the composition of rumen bacteria by three generations of full-length amplifiers sequencing in Karakul sheep over four periods with total of 72 d. 

## 2. Materials and Methods

### 2.1. Animals and Dietary Composition

All experimental procedures were approved by the Animal Research Ethics Committee of Tarim University (TLMUMO-2018-10). Twelve Karakul sheep (18 months) weighed 35 ± 3.3 kg were fitted with permanent fistula. All the animals were vaccinated against parasites before the adaption period. Sheep were fed with diets matching the standards for raising meat and sheep in the People’s Republic of China [19]. Twelve sheep were randomly assigned into four dietary NFC/NDF ratio of 0.54, 0.96, 1.37, and 1.90 as groups 1, 2, 3, and 4, respectively, with three replicates per group. All sheep were housed individually in metabolic cages (1.2 m × 1.5 m) and fed twice a day (limited-feeding) at 9:00 a.m. and 8:00 p.m. with *ad libitum* access to clean drinking water. The ingredients and nutrient level of the diet are shown in Table 1.

### 2.2. The Experimental Design and Sample Collection

The feeding experiment lasted for 72 d consisting of four periods, period I (1–18 d), II (19–36 d), III (37–54 d), and IV (55–72 d). Each period lasted for 18 d, with the first 15 d of adaption and the 3 d of sample collection. Rumen fluid was collected through rumen fistula using a vacuum pump using filter function before morning feeding from each sheep and was stored at −80 °C prior to analysis. 

### 2.3. Sample Measurements

#### DNA Extraction, PCR, and Pacio Sequencing

The total genetic DNA was extracted using QIAamp Fast DNA Stool Mini Kit (TIANGEN, Shanghai, China). The quality of the extracted DNA was evaluated by electrophoresis on 1% agarose gel. The V1–V9 regions of 16S rDNA were amplified by PCR from the extracted DNA using the universal primers: F, 5’-AGAGTTTGATCCTGGCTCAG-3’; R, 5’-GNTACCTTGTTACGACTT-3’ (synthesized by Biological engineering co., Ltd, Shanghai, China). PCR was carried out in triplicate 50-μL reactions containing 2 μL primer mix (1 μM), 1 μL gDNA (5 ng), 1 μL Trans Fastpfu, 10 μL 5× buffer, 5 μL 5× StimuLate, 5 μL dNTPs (2.5 mM each), and 26 μL NFW. Thermocycling parameters were as follows: predenaturation at 95 ℃ for 2 min; 35 cycles of denaturation at 95 ℃ for 30 s, annealing at 60 ℃ for 40 s, and extension at 72 ℃ for 90 s; and a final extension at 72 ℃ for 10 min. The PCR product was evaluated by 2% agarose gel and purified with Gel Extraction Kit (TIANGEN, Shanghai, China), and then the amplified fragments were sent to Novogene Bioinformatics Technology Co., Ltd, (Beijing, China) to be sequenced. The amplified fragments were sequenced on the Pacio platform.

### 2.4. The Sequence Analysis

All raw reads were processed to get clean reads by discarding the reads that were shorter than 1340 bp, longer than 1640 bp, or those not matching the expected barcodes. Uparse software (http://drive5.com/uparse/) [20] was used to cluster all clean reads at the similarity of 97% into operational taxonomic units (OTUs) to investigate species diversity of all the samples [21]. The OTUs were annotated by the Mothur 1.9.1 and Silva 132 (http://www.arb-silva.de/) [22] according to the reference taxonomy provided by the SSUrRNA database (http://rdp.cme.msu.edu/classifier/classifier.jsp) [23] to obtain the composition of each sample. In our experiment, Qiime pipeline 1.9.1 (http://qiime.org/) [24] was used to calculate Alpha diversity of ruminal bacteria. The Alpha diversity includes the community richness index (Chao1 and ace), community diversity index (Shannon and Simpson), and so on. 

### 2.5. Statistical Analyses

The statistical calculations were carried out using SPSS 17.0 software (IBM, New York, America). Data were normalized, and then the ANOVA test was used for group comparisons followed by the Duncan test. The *p* < 0.05 was used as a cut off criteria.

## 3. Results

### 3.1. OTUs Analysis

**Figure 1:**
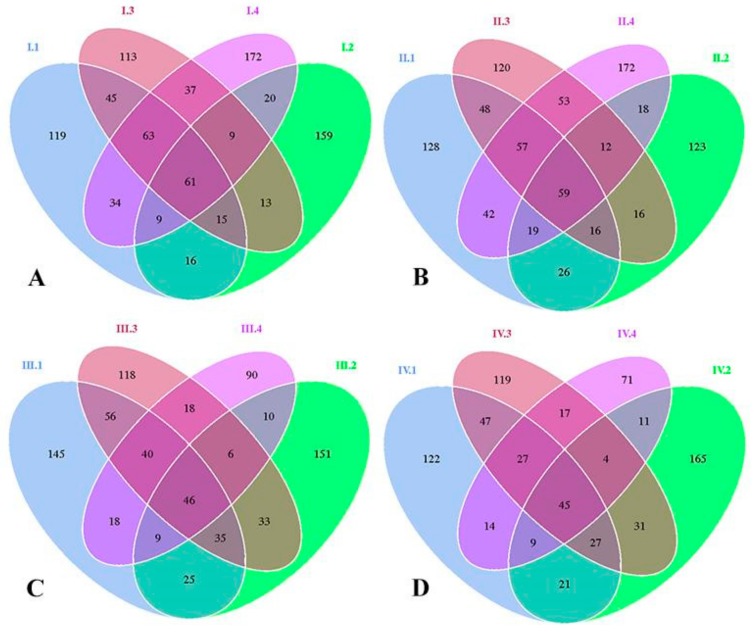
(**A–D**). Venn diagrams of operational taxonomic units (OTUs) for the four experimental periods I (1–18 d), II (19–36 d), III (37–54 d), and IV (55–72 d). Each period also shows the distribution of four groups 1, 2, 3, and 4 of Karakul sheep treated with four dietary non-fibrous carbohydrate/neutral detergent fiber (NFC/NDF) ratio of 0.54, 0.96, 1.37, and 1.90.

Venn diagrams were made to reflect the differences of bacterial species. These diagrams were formulated by analyzing common and unique OTUs among the four groups. As shown in Figure 1A–D, through four periods, the constitution of bacteria species changed with dietary NFC/NDF ratio and experimental periods. In Figure 1A, there were 362, 302, 356, and 405 OTUs in each group with unique OTUs of 119, 159, 113, and 172, respectively, for period I. In Figure 1B, groups 1, 2, 3, and 4 were with 395, 289, 381, and 432 OTUs, containing 128, 123, 120, and 172 unique OTUs, respectively, for period II. In Figure 1C, groups 1, 2, 3, and 4 were with 374, 315, 352, and 237 OTUs, containing 145, 151, 118, and 90 unique OTUs, respectively, for period III. In Figure 1D, groups 1, 2, 3, and 4 were with 312, 313, 317, and 198 OTUs, containing 122, 165, 119, and 71 unique OTUs, respectively, for period IV. 

These results showed that OTUs and unique OTUs of group 4 were decreased with the prolonged effects of the experimental periods. The quantity of OTUs and unique OTUs in group 4 were that: period II> period I> period III> period IV.

### 3.2. Alpha Diversity of OTUs

More bacterial species were found in the samples that were with a higher index of Alpha diversity. As shown in Table 2, the Alpha diversity index changed with dietary NFC/NDF ratio and experimental periods. The quantity of observed species was in the order: group 4> group 3> group 1> group 2 in the period I and II, while it was: group 1> group 3> group 2> group 4 in period III and IV, but the differences were not significant (*p* > 0.05). The index of ace in group 4 was significantly lower than in other groups in period III (*p* < 0.05), and its index in group 4 of different periods was period II> period I> period III> period IV. The index of Chao1 in group 1 was significantly higher than that in other groups in period III (*P* < 0.05), and its index in group 4 with different periods was observed as period II> period I> period III> period IV. There was no difference found in the index of Shannon and Simpson (*p* > 0.05). 

### 3.3. Effects of Dietary NFC/NDF Ratio Change on Ruminal Bacteria (Phyla)

**Figure 2:**
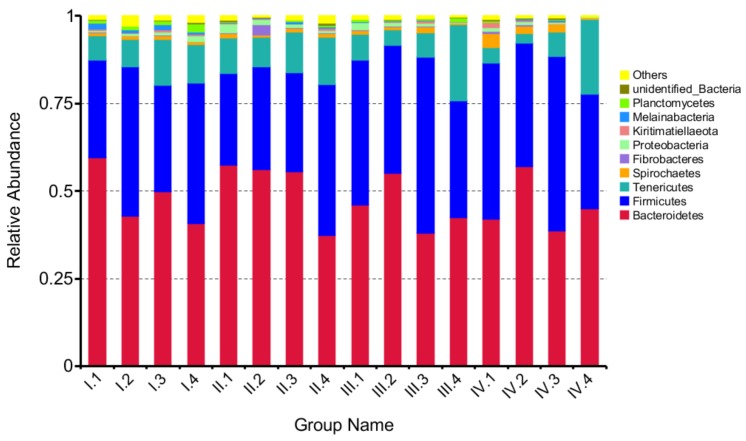
The column chart of the main dominant phyla. A color-coded bar plot, showing the bacterial phylum distribution across the different groups in different periods that were sampled. Periods I (1–18 d), II (19–36 d), III (37–54 d), and IV (55–72 d). Group 1, 2, 3, and 4 represents four groups of sheep treated with four dietary NFC/NDF ratio of 0.54, 0.96, 1.37, and 1.90, respectively.

**Figure 3:**
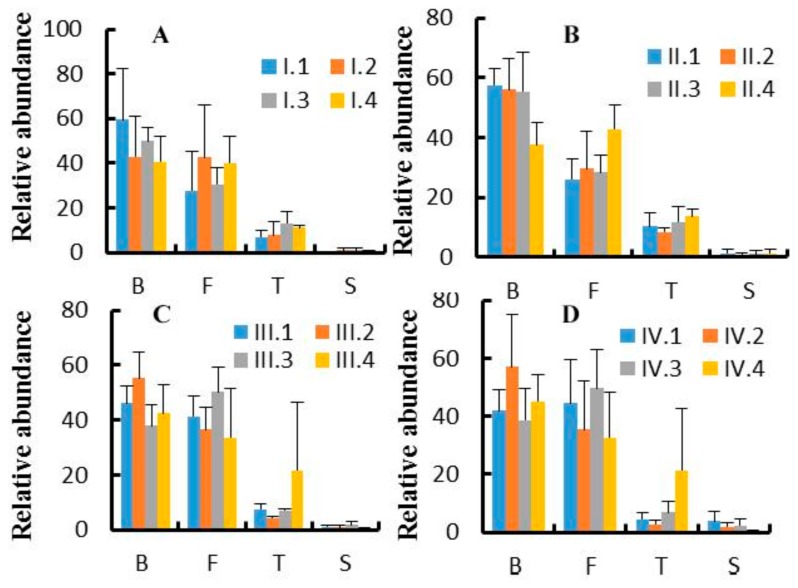
(**A**–**D**). Effects of dietary NFC/NDF ratio change on the relative abundance of phylum bacteria. Figure 3A–D represent experimental periods I (1–18 d), II (19–36 d), III (37–54 d), and IV (55–72 d). Groups 1, 2, 3, and 4 of Karakul sheep were treated with four dietary NFC/NDF ratio of 0.54, 0.96, 1.37, and 1.90. In each graph, B represents Bacteroidetes, F represents Firmicutes, T represents Tenericutes, and S represents Spirochaetes.

At the phylum level, 17 phyla were observed, out of which the top 10 phyla are shown in Figure 2. The effects of dietary NFC/NDF ratio on the relative abundance of Bacteroidetes, Firmicutes, Tenericutes, and Spirochaetes were investigated and shown in Figure 3A–D. The results showed that Bacteroidetes (37–60%) and Firmicutes (26–51%) were the main microbes in four periods, and the relative abundance of Tenericutes was around 2–22%. The relative abundance of the four phyla changed with dietary NFC/NDF ratio change and over the four periods, but there was no difference among groups and over the four periods (*p* > 0.05). 

### 3.4. Effects of Dietary NFC/NDF Ratio Change on Ruminal Bacteria (Genus)

**Figure 4:**
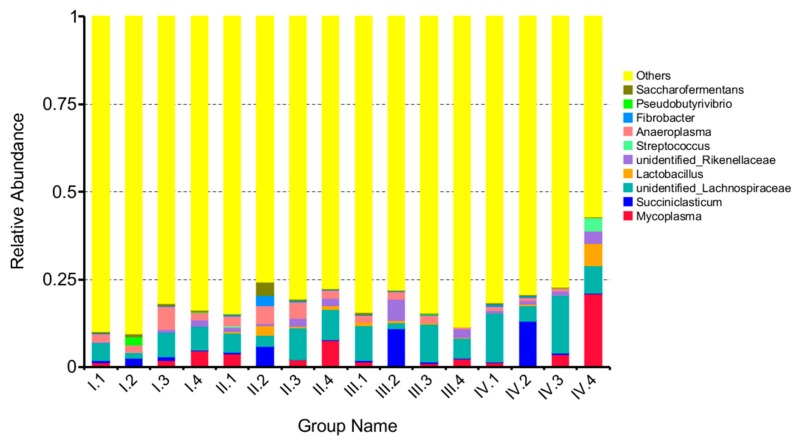
The column chart of the main dominant genera. A color-coded bar plot, showing the average bacterial genera distribution across four groups through four periods. Periods I (1–18 d), II (19–36 d), III (37–54 d), and IV (55–72 d). Groups 1, 2, 3, and 4 represent four groups of sheep treated with four dietary NFC/NDF ratio of 0.54, 0.96, 1.37, and 1.90.

**Figure 5:**
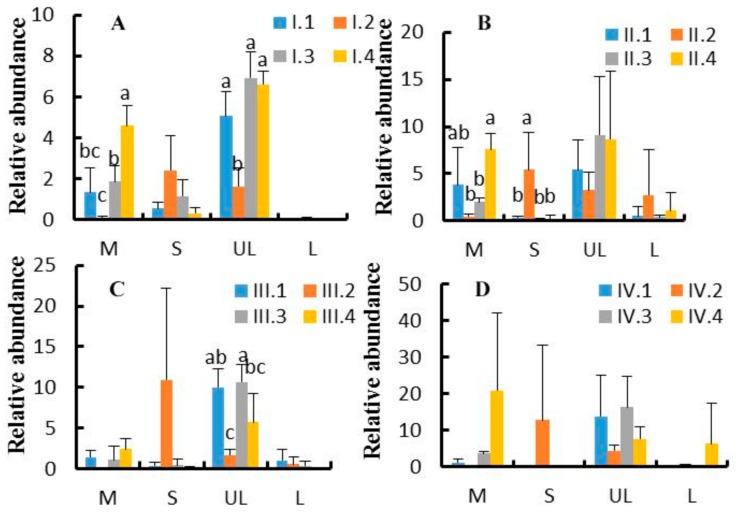
(**A**–**D**). Effects of dietary NFC/NDF ratio change on the relative abundance of ruminal genera. Figure 5A–D represent experimental periods I (1–18 d), II (19–36 d), III (37–54 d), and IV (55–72 d). Groups 1, 2, 3, and 4 represent four groups of Karakul sheep treated with four dietary NFC/NDF ratios of 0.54, 0.96, 1.37, and 1.90. In each graph, M = *Mycoplasma*; S = *Succiniclasticum*; UL = unidentified-*Lachnospiraceae*; and L = *Lactobacillus*.

At the genus level, a total of 73 genera were obtained from the sequence alignment. Figure 4 shows the top 10 genus-level information of the microbes. Effects of dietary NFC/NDF ratio change on the relative abundance of *Mycoplasma*, *Succiniclasticum*, unidentified-*Lachnospiraceae,* and *Lactobacillus* were investigated, as shown in Figure 5A–D. The results showed that unidentified-*Lachnospiraceae* (1.60–16.50%) was the main dominant genus, followed by *Mycoplasma* (0.10–22%) and *Succiniclasticum* (0.05–13.00%). The relative abundance of *Mycoplasma* in group 4 was higher than that in group 2 in the period I and II (*p* < 0.05). The relative abundance of *Succiniclasticum* in group 2 was higher than the other groups in period II (*p* < 0.05). The relative abundance of unidentified-*Lachnospiraceae* was ranked as group 3> group 4> group 1> group 2 in the period, I.; while it was ranked as group 3> group 1> group 4> group 2 in period III, with significant differences (*p* < 0.05). The relative abundance of *Lactobacillus* was low, and no significant difference was found across treatments (*p* > 0.05). 

### 3.5. Effects of Dietary NFC/NDF Ratio Change on the Relative Abundance of Cellulose, Semi-cellulose Degrading Bacteria, and Starch Degrading Bacteria (Species)

**Figure 6:**
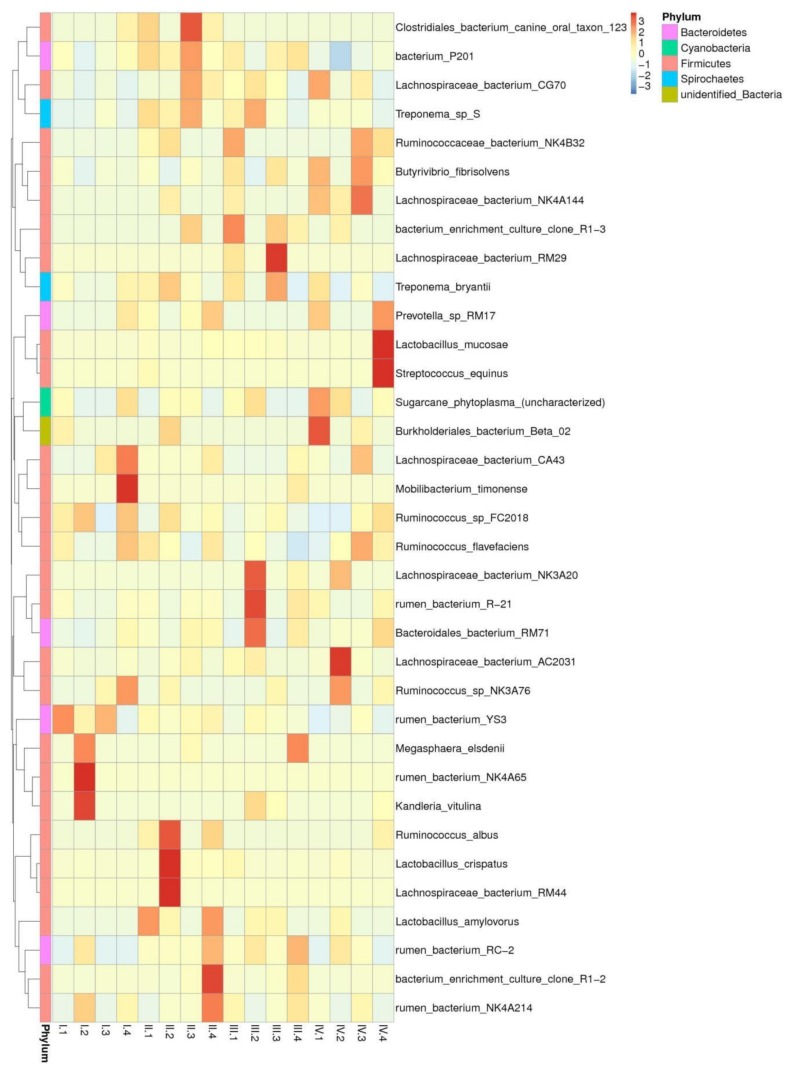
The relative abundance cluster map of species. Sample information is listed in the longitudinal direction, and species annotated information is listed in the crosswise direction. The species cluster tree is shown on the left-hand side, and the corresponding values of the intermediate heat map are the Z values obtained after the relative abundance of each row of species, which has been standardized.

A total of 78 species were detected in our study, out of which 35 are shown in Figure 6. The cellulose-degrading bacteria that were detected included *Butyrivibrio-fibrisolvens* and *Ruminococcus-flavefaciens*, the semi-cellulose-degrading bacteria included *Lachnospiraceae-bacterium-AC2031* and *Lachnospiraceae-bacterium-NK3A20*, and the starch-degrading bacteria included *Streptococcus-equinus* and *prevotella-sp-RM17*. As shown in Table 3, the relative abundance of *Butyrivibrio-fibrisolvens* was 0.60–14.50%, which was ranked in different groups as group 3> group 1> group 4> group 2 in period III, whereas it was ranked as group 1> group 3> group 4> group 2 in period I, with significant differences (*p* < 0.05). The relative abundance of *Ruminococcus-flavefaciens* was not different across the treatments (*p* > 0.05). The relative abundance of *Lachnospiraceae-bacterium-AC2031*, *Lachnospiraceae-bacterium-NK3A20*, *Streptococcus-equinus,* and *prevotella-sp-RM17* was low, with no significant differences across the treatments (*p* > 0.05).

## 4. Discussion

### 4.1. Effects of Dietary NFC/NDF Ratio Change on Rumen Bacteria in Karakul Sheep

The dietary NFC/NDF ratio had major effects on the rumen microbial community in this study. It has been shown that the number and diversity of rumen bacteria in goats fed with a high grain diet (71.5%) were lower than those fed with a high forage diet (0% grain) [25]. Mu et al. [26] showed that feeding more buckwheat straw resulted in decreased bacterial diversity. In another study, Ji et al. [27] showed that the population of rumen bacteria in lambs fed with a high concentrate diet was lower than those fed with a high forage diet. In our study, it was observed that the rumen bacterial diversity in Karakul sheep decreased in the diet with the highest carbohydrate (NFC/NDF ratio of 1.90) with the prolonged experimental period. This was likely due to the fact that a high dietary NFC/NDF ratio was degraded rapidly in the rumen, and the stable environment of rumen bacteria was interrupted, leading to the decreased diversity of bacteria. 

Research showed that diet with easily fermentable carbohydrates would decrease fiber degradation [28], resulting in the imbalance of cellulolytic bacterial species. In our experiment, the change of Bacteroidetes and Firmicutes were investigated, as Bacteroidetes are of great importance in non-fibrous degradation, and Firmicutes are involved in fiber degradation [29]. A large number of studies have shown that Bacteroidetes and Firmicutes are the most dominant flora in the gastrointestinal tract of mammals [30,31,32,33]. Li et al. [34] found that when the calves were fed with two different dietary NFC/NDF ratios, Bacteroidetes and Firmicutes were still the main dominant flora. In our experiment, Bacteroidetes and Firmicutes were also the main dominant phyla regardless of what ratio of the dietary NFC/NDF were offered, despite research showed that Bacteroidetes was directly related with fiber substances, and the relative abundance of it was closely related to fiber degradation [35]. Some studies revealed that the abundance of bacteria in the same sample would be different if the gene region is sequenced differently [36]. This might partly explain the discrepancy we observed in this study. In our study, the region V1–V9 was sequenced, and the results indicated that dietary NFC/NDF ratio had no effects on the relative abundance of Bacteroidetes. However, Kim et al. [37] investigated the rumen bacteria of beef cattle by sequencing the V1–V3 region, and the results showed that Bacteroidetes contents in high forage groups were lower than that in high proportions of the cereal group. This difference could be due to the disparity of species and the sequencing regions measured. Therefore, the interpretation of reported results should take consideration of methods and sequencing regions measured in different studies. In our study, the relative abundance of Firmicutes tended to be higher in the dietary NFC/NDF ratio of 1.37, and the relative abundance of unidentified-*Lachnospiraceae* was higher than the relative abundance of NFC/NDF ratio of 1.37. Thus, a dietary NFC/NDF ratio of 1.37 might have a better fiber degradation rate, which needs to be further investigated. *Lactobacillus* can ferment sugars in the diet and produce acid, which would decrease ruminal pH. In our study, the relative abundance of *Lactobacillus* was increased in dietary NFC/NDF ratio of 1.90 in period IV, and the results might indicate that a higher NFC/NDF ratio enhanced the relative abundance of acid-forming bacteria, which might have decreased the diversity of ruminal bacteria in Karakul sheep.

### 4.2. Effects of Dietary NFC/NDF Ratio Change on Cellulose-Degrading Bacteria, Semi-Cellulose-Degrading Bacteria, and Starch-Degrading Bacteria

Bacteria and fungi play a vital role in the decomposition and utilization of cellulose, especially the cellulose-degrading bacteria, like *Ruminococcus-flavefaciens*, *Fibrobacter-succinogenes*, *Butyrivibrio-fibrisolvens,* and *Clostridium*. Semi-cellulose-degrading bacteria like *Lachnospira* and *Succinivibrio* could degrade semi-cellulose. In our study, the relative abundance of main cellulose-degrading bacteria was observed with the highest in dietary NFC/NDF ratio of 1.37. Zened et al. [38] showed that the relative abundance of cellulose-degrading bacteria was lower in quantity. In the current study, the relative abundance of *Ruminococcus-flavefaciens* was lower, along with semi-cellulose-degrading bacteria.

The main starch-degrading bacteria include *Streptococcus,* unidentified-*Prevotellaceae,* and *Ruminobacter.* Starch content in NFC is an important factor affecting dry matter intake and digestibility. Oba et al. [39] reported that the dry matter intake of dairy cows increased when lactating cows were fed additional grains. In another study, Huo et al. [40] showed that the contents of *Prevotella* increased in goats after feeding hay diet. However, in the current study, the relative abundance of *Streptococcus-equinus* and *prevotella-sp-RM17* was lower, with no significant effects, which might be due to the reason that only rumen fluid was examined here. In addition, the relative abundance of main starch-degrading bacteria was higher in dietary NFC/NDF ratio of 1.90 in this study. 

### 4.3. The Limitation of This Study

It is very important to note that most of the treatment effects were non-significant on rumen microbial diversity and population. We believe this is partly due to the low replication units we had examined in the study and partly due to the large environmental variations between individual sheep. Nevertheless, the study revealed some interesting results as discussed above and confirmed the number of observations, as described in the previous studies. Future work using three generations of full-length amplifiers sequencing should be targeted to use more replications and perhaps with sheep sources from a more controlled environment and with a similar feeding history.

## 5. Conclusions

The study concluded that rumen bacteria diversity was decreased in dietary NFC/NDF ratio of 1.90 with the prolonged effects of the experimental period. The most dominant phyla in Karakul sheep rumen didn’t change with the dietary NFC/NDF ratio. The relative abundance of unidentified-*Lachnospiraceae* and main cellulose-degrading bacteria was observed higher in dietary NFC/NDF ratio of 1.37.

## Figures and Tables

**Table 1:** The ingredients and nutrient composition of the diet (% of dry matter).

Ingredients/Nutrient Levels ^1^	1	2	3	4
Cotton seed hulls	30	20	15	13
Alfalfa grain	33	30	25	24
Corn	20	35.40	45	54
Bean pulp	2	2	2	2
Wheat bran	12.70	10.30	10.70	4.70
NaCl	0.80	0.80	0.80	0.80
CaCO_3_	0.50	0.50	0.50	0.50
Premix ^2^	1	1	1	1
Total	100	100	100	100
Dry matter	95.54	95.74	96.30	95.55
Crude protein	14.76	14.96	14.10	13.43
Ether Extract	2.08	2.11	2.33	2.58
Ash	8.85	7.62	6.86	6.35
Ca	0.74	0.73	0.72	0.75
P	0.26	0.25	0.24	0.22
NFC ^3^	26.11	36.98	44.41	50.84
NDF	48.20	38.33	32.30	26.80
ADF	31.75	28.73	23.80	21.41
NFC/NDF ratio	0.54	0.96	1.37	1.90

^1^ Nutrition level, except NFC, was measured value. ^2^ The premix provided the following per kg of diets: VA_1_ 800 IU, VD_3_ 600 IU, VE 30 mg, Fe 65 mg, Se 0.15 mg, I 0.6 mg, Cu 10 mg, Mn 28 mg, Zn 45 mg, Cu 12 mg. ^3^ NFC=(1−NDF−CP−Fat−Ash) × 100%. NFC: non-fibrous carbohydrate; NDF: neutral detergent fiber.

**Table 2:** Analysis of Alpha diversity at 0.03 distance.

Periods	Groups	Observed Species	Shannon	Simpson	Chao1	Ace
I	1	165	4.76	0.79	322.48	362.77
	2	138	4.86	0.86	269.37	308.97
	3	172	5.97	0.94	237.43	278.69
	4	201	6.27	0.96	371.14	423.93
	SEM	10.26	0.29	0.03	23.20	26.16
	*p*-value	0.186	0.153	0.141	0.179	0.221
II	1	176	5.61	0.92	342.79	384.79
	2	141	5.15	0.90	227.96	276.78
	3	184	5.94	0.93	357.81	382.58
	4	212	6.55	0.98	409.78	475.55
	SEM	11.50	0.25	0.01	29.46	32.07
	*p*-value	0.19	0.25	0.39	0.16	0.18
III	1	170	5.71	0.95	352.03	407.15
	2	144	5.32	0.92	224.82	259.23
	3	164	5.52	0.91	188.22	301.56
	4	111	4.50	0.86	238.08	227.61
	SEM	8.79	0.21	0.02	22.15	24.22
	*p*-value	0.06	0.16	0.35	0.02	0.02
IV	1	145	5.23	0.91	238.08	256.55
	2	137	5.22	0.92	306.31	309.69
	3	143	5.31	0.92	250.78	294.17
	4	98	4.54	0.88	170.44	183.50
	SEM	8.13	0.19	0.02	21.46	20.47
	*p*-value	0.11	0.52	0.79	0.16	0.11

Note: Period I (1–18 d), II (19–36 d), III (37–54 d), and IV (55–72 d). Groups 1, 2, 3, and 4 represent four groups of Karakul sheep treated with four dietary NFC/NDF ratios of 0.54, 0.96, 1.37, and 1.90, respectively.

**Table 3:** Effects of dietary NFC/NDF ratio change on the relative abundance of cellulose-degrading bacteria, semi-cellulose-degrading bacteria, and starch-degrading bacteria in Karakul Sheep.

Periods	Groups	*Butyrivibrio-fibrisolvens*	*Ruminococcus-flavefaciens*	*Lachnospiraceae-bacterium-AC2031*	*Lachnospiraceae-bacterium-NK3A20*	*Streptococcus-equinus*	*Prevotella-ruminicola*
I	1	3.64	0.30	0.05	-	-	-
	2	0.66	0.15	-	-	0.05	-
	3	3.23	0.15	-	-	-	-
	4	2.63	0.45	0.10	-	-	0.10
	SEM	0.386	0.07	0.03	-	0.02	0.03
	*p*-value	<0.01	0.36	0.56	-	0.44	0.44
II	1	3.99	0.35	-	-	0.45	0.05
	2	0.45	0.25	0.10	-	-	-
	3	4.24	0.10	0.25	-	-	0.05
	4	2.53	0.35	-	-	-	0.15
	SEM	2.26	0.06	0.04	-	0.11	0.04
	*p*-value	0.13	0.38	0.06	-	0.44	0.65
III	1	8.84	0.15	0.20	-	-	-
	2	0.35	0.25	0.30	0.25	-	-
	3	8.89	0.15	-	-	-	-
	4	3.23	0.05	-	0.05	-	-
	SEM	1.20	0.05	0.05	0.06	-	-
	*p*-value	<0.01	0.67	0.11	0.53	-	-
IV	1	12.42	0.10	0.05	-	0.05	0.15
	2	2.53	0.25	1.16	0.15	-	-
	3	14.34	0.51	0.10	-	-	-
	4	5.25	0.30	-	-	3.89	0.2
	SEM	2.32	0.06	0.19	0.04	0.98	0.05
	*p*-value	0.22	1.14	0.055	0.44	0.45	0.42
